# Study of the influence of spatial effects on the ground surface and buildings in a two-lane tunnel boring

**DOI:** 10.1016/j.heliyon.2024.e37667

**Published:** 2024-09-11

**Authors:** Zhiqiang Chen, Wenlong Xiang, Zhaojian Hu, Mingjin Li, Jintao Wang, Dongxiang Hou, Zhen Huang

**Affiliations:** a China State Construction Engineering Corporation Infrastructure Construction Investment CO. LTD., Wuhan, 430073, China; b China Construction Third Bureau Group CO. LTD., Wuhan, 430073, China; c School of Civil Engineering and Architecture, Guangxi University, Nanning, 530004, China

**Keywords:** Shield tunnel, Double-lane boring, Construction process, Building deformation, Numerical simulation

## Abstract

There are a series of engineering risks, such as ground subsidence, building tilt and cracking, in the process of shield tunnel driving through buildings, which have many adverse effects on urban residents and engineers. In particular, the differences in the effects of the interaction of two-lane tunnels on the building structure and ground deformation field are less often considered under different spatial effects such as construction sequences and tunnel spacings. As well as the problem of analyzing the building as a whole out of reality. To solve these problems, the spatial effect of the shield tunnel underpassing the shallow foundation building is simulated by Plaxis3D software to study the sensitivity analysis of surface settlement and the internal forces and deformation law of the building above in the process of tunneling underpassing with different depths of burial *H* and horizontal distance *D* of the double line tunnel. The engineering impact zoning method can investigate the safety of tunnels and buildings under different spatial effects when tunnels pass through buildings. The splitting of the building into plates and columns can reveal the forces and deformation laws of different structural parts. The results show that during the construction process of the double-line tunnel, the tunnel constructed first has a “blocking effect” on the tunnel constructed later, which affects the distribution of the disturbance area to a certain extent and changes the curve shape of the settlement trough. When the “blocking effect” occurs, the surface settlement and building deformation will be significantly reduced. In the internal forces of the building, the plate structure is mainly subjected to changes in axial forces, while the column structure is mainly affected by shear forces and bending moments. The factor of safety of tunnels decreases as the tunnel spacing decreases and as the building loads above increase.

## 1 Introduction

As a modern tunneling method, shield tunneling, compared with traditional drill blasting methods and open cut methods, has the advantages of high precision controllability, excellent geological adaptability, maximized space utilization, and low environmental impact [[1], [2], [3]]. However, new tunnels inevitably cause ground loss and damage and deformation of existing building complexes and urban infrastructures due to the limitations of urban underground space. At the same time, the presence of existing buildings increases the magnitude of the upper loads on the tunnel structure, and tunnel excavation can cause damage and impairments to the surrounding environment and structures, such as surface subsidence [4,5], stress on the foundations of buildings [6], structural damage [7], vibrations [8], and changes in the groundwater level [9], which bring great challenges and risks to construction, affecting the durability and safety of tunnels.

To minimize these problems, many scholars perform reliable damage prediction of tunnels and adjacent structures before tunnel excavation [10]. Ninić et al. [11] proposed a prototype concept for real-time support of steering of tunnel boring machines based on simulation and real-time monitoring. Cao et al. [12] proposed a real-time ground settlement prediction and tunnel boring machine operation parameter adjustment modelling method by combining artificial neural networks instead of time-consuming finite element simulation, while Gong et al. [13] proposed a computational method for the ground deformation of shield tunnels under multistory buildings based on the Mindlin solution promoted in a semi-infinite space. Sirivachiraporn et al. [14] based on shield excavation monitoring data to analyse the shield pressure and pile foundation in a thick soft clay layer to evaluate the ground motion characteristics and response of adjacent buildings.

However, the above methods require a large amount of training data and monitoring data, which are difficult to obtain or incomplete, resulting in lower accuracy and generalization ability of the model. Physical models are easy to understand and validate due to the similarity of morphological structures to the characteristics and nature of the real thing, while numerical simulations can more realistically represent structure-soil interactions, and the deformation and internal forces changes are easy to observe, so the two methods have become commonly used tools in the design stage of tunnel engineering. Zhu et al. [15] investigated the displacement response of pile-soil in close proximity to existing pile foundations during shield tunnel construction. Xu et al. [16] conducted a series of model tests and field investigations to simulate earth pressure balance (EPB) shield machine tunneling in soft soil foundation, which revealed the intrinsic relationship between machine operating parameters and soil disturbance. Wu et al. [17] analysed the changes of internal forces and displacement of bar foundation, and came up with optimal tunneling parameters for different types of shield machines through numerical simulation and field monitoring data. Meschke et al. [18] established a finite element model of the interaction between mechanized tunnel construction and the soil around the ground and the existing pile foundation building to study the relevant interaction processes in urban tunnel excavation. Guan et al. [19] proposed a reasonable freezing method and parameters, and verified through numerical simulation that the method effectively reduces tube sheet deformation, surface settlement and building vertical displacement. Li et al. [20] proposed a new combined reinforcement method of sloped tube roof grouting to study the deformation response of three reinforcement schemes of overlapping tunnels underneath an old destabilized building. Zheng et al. [21] used a combination of example analysis and numerical simulation to study the impact of shield tunnel excavation on pile buildings in soft ground, and the load transfer mechanism of pile foundations was comprehensively analysed.

As can be seen from the existing studies, researchers usually take the influence of single-lane tunnel boring as the research basis, and at the same time, a large number of studies have been carried out on the influence of shield tunnels on existing tunnels [[22], [23], [24]], bridges [[25], [26]], and railroads [27]. However, on the one hand, the existing studies neglected the possible structural cracking and tilting problems of buildings under shield tunnels boring; on the other hand, they only considered the impact of tunnels on pits or viewed the buildings as a whole to be analysed, while the internal structure and material nature differences of the buildings need to be taken into account in the actual situation. In the actual shield tunnel excavation process, which is mainly dominated by two-lane tunnels, the selection of spatial effect factors such as excavation depth, excavation location, tunnel spacing, construction sequence, etc. of the double tunnels will greatly affect the quality of the project and the safety of the building. Therefore, the study problem of this paper is to investigate the influence of the spatial effect of the two-lane shield tunnel excavation process on the surface settlement around the building, the deformation of the building structure and the change of the internal forces, to assess the safety influence range of each construction plan, so as to choose a reasonable construction layout in to avoid the damage of the building and the ground subsidence problem brought by the excavation process.

To address the above issues, this study adopts the numerical software Plaxis to construct a refined tunnel construction model with differences in building materials and actual ground parameters, to investigates the deformation and force of the surrounding ground surface and building when a two-lane tunnel at different spatial locations passes under a shallow foundation building. The research process and purposes are divided into two steps: (1) establishing two-lane tunnel models with different construction sequences (sequential construction, simultaneous construction) and different spatial locations (horizontal distance *D* and burial depth *H*). These models are used to investigate the surface settlement sensitivity, building settlement and deformation, and structural internal forces during the tunnel boring process of the two-lane shield tunnels. The results of the study can provide references for construction of the two-lane tunnel, such as optimizing the construction plan and the building safety warning plan, selecting reasonable construction routes, shield tunnel spacing and depth of burial, and proposing effective measures to reduce the deformation and damage of the building. (2) The impact zoning study on the tunneling process of two-lane shield tunnels passing through buildings was carried out to calculate the safety coefficients of the tunnels under different building loads, and compare them with the safety coefficients under no building loads to find the ratio of the safety coefficients, so as to assess the safety situation under each working condition.

## 2 Numerical simulation

### 2.1 Model building

**Fig. 1:**
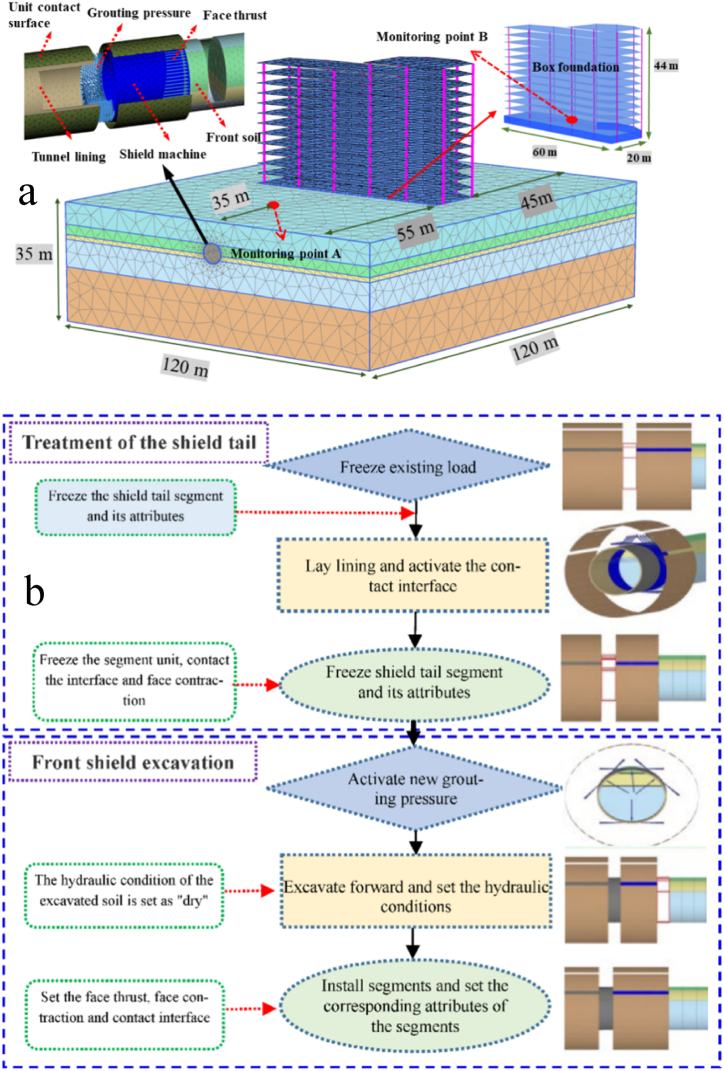
Construction of the numerical model and simulation of shield tunneling process: (a) The refined numerical model; (b) The simulation of shield tunneling process.

In this study, a project subway shield section was used as a study case. The building is a 13-story plate-column structural system with a box foundation as the building foundation type, and the diameter of the shield construction underneath is 6 m. The numerical model of the shield tunnel under the building and its running program are shown in Fig. 1. It is clear from Fig. 1(a) that the dimensions of each part of the model and their spatial location, and Fig. 1(b) shows the operation flow of the excavation process. In this, the frame structure of the building was simulated by plate elements and beam elements. A large number of this building type exists in the city. The selection of this building can more realistically reflect the situation in the actual engineering environment, which helps to improve the applicability and generalizability of the research results. Finite element software Plaxis3D was used for numerical modelling and analysis, the basic simulation principle of this software is to discretize the continuous solution area into a finite set of units and meshes, which are combined together in a certain way. Appropriate interpolation functions are chosen according to the characteristics of the soil structure, and its properties are used to find out the unit stiffness matrix, aggregate all the units to get the overall equilibrium equations, and derive the displacement and internal forces at any point from the known displacements and internal forces. Plaxis 3D is widely used in the field of geotechnical engineering, has modules for shield excavation, and can also construct surface buildings. Therefore, it is appropriate to use Plaxis 3D for the numerical simulation of this project.

### 2.2 Constitutive and parameter determination

#### 2.2.1 Constitutive model

The soil hardening constitutive model is selected to simulate the stress‒strain characteristics of soil. This model belongs to the second-order advanced constitutive model, which can describe the irreversible strain caused by the principal partial loading. At the same time, considering the dilatation of soil and the difference in the loading and unloading/reloading modulus of soil, it is suitable for clay, silty soil, sandy soil and gravel soil.

In the principal stress space, the complete expression of the shear yield surface of the soil hardening model (with pressure as positive) is [28]:

(1) $$\begin{array}{l} F_{\text{SI}}=\frac{1}{E_{50}}\frac{\left(\sigma_{1}-\sigma_{3}\right)}{1-\left(\sigma_{1}-\sigma_{3}\right)/q_{a}}-\frac{2}{E_{\text{ur}}}\left(\sigma_{1}-\sigma_{3}\right)-\gamma_{s}^{p}\\ F_{\text{SII}}=\frac{1}{E_{50}}\frac{\left(\sigma_{1}-\sigma_{2}\right)}{1-\left(\sigma_{1}-\sigma_{2}\right)/q_{a}}-\frac{2}{E_{\text{ur}}}\left(\sigma_{1}-\sigma_{2}\right)-\gamma_{s}^{p}\\ F_{\text{SIII}}=\frac{1}{E_{50}}\frac{\left(\sigma_{2}-\sigma_{3}\right)}{1-\left(\sigma_{2}-\sigma_{3}\right)/q_{a}}-\frac{2}{E_{\text{ur}}}\left(\sigma_{2}-\sigma_{3}\right)-\gamma_{s}^{p} \end{array}\}$$

where *E*_*50*_ and *E*_*ur*_ are the secant modulus of loading and the unloading/reloading modulus when the confining pressure is σ_3_, respectively, and *γ*_s_^p^ is the plastic shear strain on the shear yield surface. *q*_*a*_ is the limit deviatoric stress.

The expressions for *E*_*50*_ and *E*_*ur*_ of Eq. (1) are given by Eq. (2):

(2) $$\begin{array}{l} E_{50}=E_{50}^{\text{ref}}{\left(\frac{\sigma_{3}\;\sin\;\varphi +c\;\cot\;\varphi }{\sigma^{\text{ref}}\;\sin\;\varphi +c\;\cot\;\varphi }\right)}^{m}\\ E_{\text{ur}}=E_{\text{ur}}^{\text{ref}}{\left(\frac{\sigma_{3}\;\sin\;\varphi +c\;\cot\;\varphi }{\sigma^{\text{ref}}\;\sin\;\varphi +c\;\cot\;\varphi }\right)}^{m} \end{array}\}$$

where *E*_*50*_^*ref*^ and *E*_*ur*_^*ref*^ are the reference values of the loading modulus and unloading/reloading modulus, respectively, under a given confining pressure (*σ*_*ref*_) and *φ* is the friction angle of the soil. *c* is cohesion; *m* is the power index obtained by fitting the triaxial test results.

The expression for *q*_a_ of Eq. (1) is given by Eq. (3):

(3) $$q_{a}=\frac{q_{f}}{R_{f}}=\frac{1}{R_{f}}\left(c\;\cot\;\varphi +\sigma_{3}\right)\frac{2\;\sin\;\varphi }{1-\sin\;\varphi }$$

where *q*_f_ is the limiting bias stress calculated by the Moore-Coulomb criterion. The expression for *q*_f_ of Eq. (3) is given by Eq. (4).

(4) $$q_{f}=\left(2\;c\;\cos\;\varphi +2\sigma_{3}\;\sin\;\varphi \right)/\left(1-\sin\;\varphi \right)$$

When the partial stress is *q* < *q*_f_, the soil is in the elastic deformation stage.

In Eq. (3): R_f_ is the destruction ratio, and it is expressed as in Eq. (5).

(5) $$R_{f}={\left(\sigma_{1}-\sigma_{3}\right)}_{f}/{\left(\sigma_{1}-\sigma_{3}\right)}_{\text{ult}}=q_{f}/q$$

where the subscript “ult” represents the limit and “f" represents the damage.

The increment of *R*_f_ is expressed as in Eq. (6).:

(6) $$d\gamma_{S}^{p}=d\varepsilon_{1}^{p}-d\varepsilon_{2}^{p}-d\varepsilon_{3}^{p}$$

where *ε*_1_^p^, *ε*_2_^p^, and *ε*_3_^p^ are the first, second and third principal strains of plasticity, respectively.

Through the above formula, the limit deviatoric stress *q*_a_ can be obtained. When *q* > *q*_f_, the soil enters the plastic deformation stage. With the change in hardening parameters, the yield surface of the soil hardening model also changes.

#### 2.2.2 Model parameters

According to the typical stratigraphy of the project area given in the literature [[29], [30], [31], [32]], the stratigraphic parameters are shown in Table 1. The relevant parameters of the shield tunnel and buildings are shown in Table 2.

**Table 1:** Stratigraphic calculation parameters.

Layer	Thickness/*d* (m)	Unit weight /*γ* (kN·m^−3^)	$E_{50}^{\text{ref}}$(kN·m^−2^)	$E_{\text{oed}}^{\text{ref}}$(kN·m^−2^)	$E_{\text{ur}}^{\text{ref}}$(kN·m^−2^)	Cohesion/*c* (kN·m-2)	Angle of internal friction/*φ*(°)	*V*_*ur*_
Plain fill	6.45	17.8	11.25 × 10^3^	9 × 10^3^	55.56 × 10^3^	5	25	0.25
Mucky clay	3	19	20 × 10^3^	25.61 × 10^3^	94.84 × 10^3^	10	18	0.2
Clay	1.4	19.7	13.5 × 10^3^	13.5 × 10^3^	40.5 × 10^3^	27	22	0.2
Plastic oak Sandy clay	8.1	18.3	30 × 10^3^	36 × 10^3^	110.8 × 10^3^	5	25	0.2
Weathered rock	16.05	20	15 × 10^3^	15 × 10^3^	45 × 10^3^	25	32	0.2

Note: In the table, *E*ref oed is the Tangent stiffness of confined compression test, *m* is the Stress-dependent power exponent of stiffness, and *V*_*ur*_ is the Poisson ratio.

**Table 2:** Material parameters.

material	Severe/γ (kN/m^3^)	Modulus of elasticity/E (kN/m^2^)	Thickness/d (m)	Poisson's ratio/v
lining	27	27.6 × 10^6^	0.3	0.167
Shield machine plate shell floor	120	23 × 10^6^	0.3	0
floor	25	30 × 10^6^	0.1	0
Foundation sidewall	25	30 × 10^6^	0.2	0
Foundation floor	25	30 × 10^6^	0.25	0
beam	25	30 × 10^6^	/	/
Independent foundation	25	30 × 10^6^	1.5	0

#### 2.2.3 Shield tunnelling parameters

① Supporting force of the working face

The three-factor method commonly used in foundation bearing capacity analysis is used to determine the limiting support pressure, and the formula is as follows [33]:

(7) $$\sigma_{t}=cN_{c}+\gamma DN_{\gamma }+qN_{q}$$

In Eq. (7), *N*_*c*_, *N*_*γ*_, and *N*_*q*_ represent the coefficients of influence produced by cohesion *c*, gravity *γ* and overlying load *q* on the ultimate support pressure, respectively.

The minimum supporting reaction of the palm surface is 142 kN/m^2^ when *H* = 6 m and 192.5 kN/m^2^ when *H* = 18 m. For ease of calculation, when *H* = 6 m, the support force of the tunnel face linearly increases from 150 to 240 kN/m^2^ from top to bottom. When *H* = 18 m, the support force of the tunnel face is a linear load increasing from 260 to 350 kN/m^2^.

② Grouting pressure

Grouting pressure has a significant effect on the bearing capacity of the tunnel structure and surface settlement. The formula for the critical value of grouting pressure above and below is as follows:

(8) $$\begin{array}{l} P_{\text{up}}=\gamma h^{*}\;\tan\left(\frac{\pi }{4}+\frac{\varphi }{2}\right)+2c\;\tan\left(\frac{\pi }{4}+\frac{\varphi }{2}\right)\\ P_{\text{ma}}=\gamma h^{*}\;\tan\left(\frac{\pi }{4}-\frac{\varphi }{2}\right)-2c\;\tan\left(\frac{\pi }{4}-\frac{\varphi }{2}\right) \end{array}\}$$

In Eq. (8), γ is the soil bulk density; h* is the buried depth of the grouting hole; and *P*_up_ and *P*_ma_ are the upper and lower critical values of the grouting pressure.

In order to facilitate the calculation, the grouting pressures in the tunnel vault at *H* = 6m, 9m, 12m, 15m and 18m are 120 kN/m^2^, 160 kN/m^2^, 200 kN/m^2^, 250 kN/m^2^ and 300 kN/m^2^ respectively, as the depth of the grouting point increases, the grouting pressure increases at a constant rate of 15 kN m^−2^/m. The calculation results and numerical values are shown in Table 3.

### 2.3 Simulation scheme

**Fig. 2:**
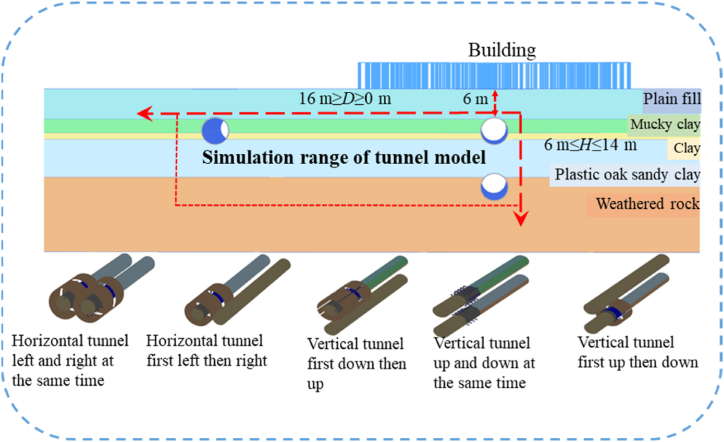
Simulation scheme.

The tunnel geometric model was divided into several construction sections along the axis of the tunnel, each section 2 m long, and the shield tunneling machine drove from the front of the formation model until penetrating the entire model. The simulation scheme is divided into a horizontal double-track tunnel and a vertical double-track tunnel. One tunnel is fixed at the buried depth *H* = 6 m under the building, the horizontal spacing between the tunnel and the other tunnel is set as *D* = 4 m, 8 m, 12 m and 16 m, and the vertical spacing is *h* = 2 m, 4 m, 6 m and 8 m. Simulate the construction sequence of five kinds of double-line tunnels, namely, the existing tunnel construction on the upper side, the existing tunnel construction on the lower side, the simultaneous construction of the upper and lower double-line tunnels, the existing tunnel construction on the left (right) side, and the simultaneous construction of the left and right double-line tunnels. The specific simulation scheme is shown in Fig. 2. Meanwhile, schemes simulating different buried depths *H* of a single-line tunnel and different horizontal distances *D* from the center of the building are used for comparative analysis. The buried depths *H* are 6 m, 9 m, 12 m, 15 m, 18 m, respectively, and the horizontal distances *D* between the center of the tunnel and the center of the building are 0 m, 5 m, 10 m, 15 m, 20 m, 25 m, 30 m, 35 m, 40 m, respectively.

### 2.4 Model validation

**Fig. 3:**
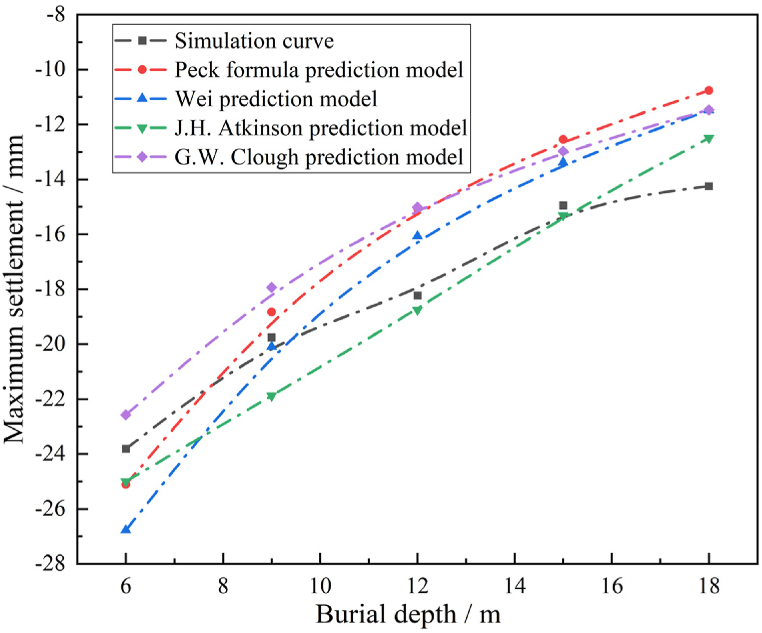
The calculated value and theoretical solution of the maximum surface subsidence.

The calculated settlement values in this paper are compared with the theoretical solutions [[34], [35], [36], [37]]. The maximum surface settlement values calculated for different burial depths *H* are shown in Fig. 3.

As seen from Fig. 3, the shapes of all curves in the figure are similar, and the calculated maximum settlement values of different burial depths are in good agreement with the calculated results of other prediction models. When the burial depth is less than 6 m, the tunnel passes through the stratum mainly in the area of artificial foundation soil. At depths of 9 m–16 m, the strata are dominated by different types of soils. When *H* > 16 m, strata is simulated through linear elastic constitutive model which also leads to a sudden change in the slope of the curve of the numerical calculation value at buried depths *H* = 9 m and 16 m.

## 3 Interpretation of results

### 3.1 Sensitivity analysis of surface subsidence

#### 3.1.1 Law of surface axial settlement

In order to investigate the effect of the two-lane shield tunnel at different spatial locations on the surface settlement S, point A shown in Fig. 1(a) was selected for sensitivity analysis. The surface settlement value was extracted every 3 m on the section of point A, and the surface settlement sensitivity under different working conditions was explored by changing different spatial positions.

**Fig. 4:**
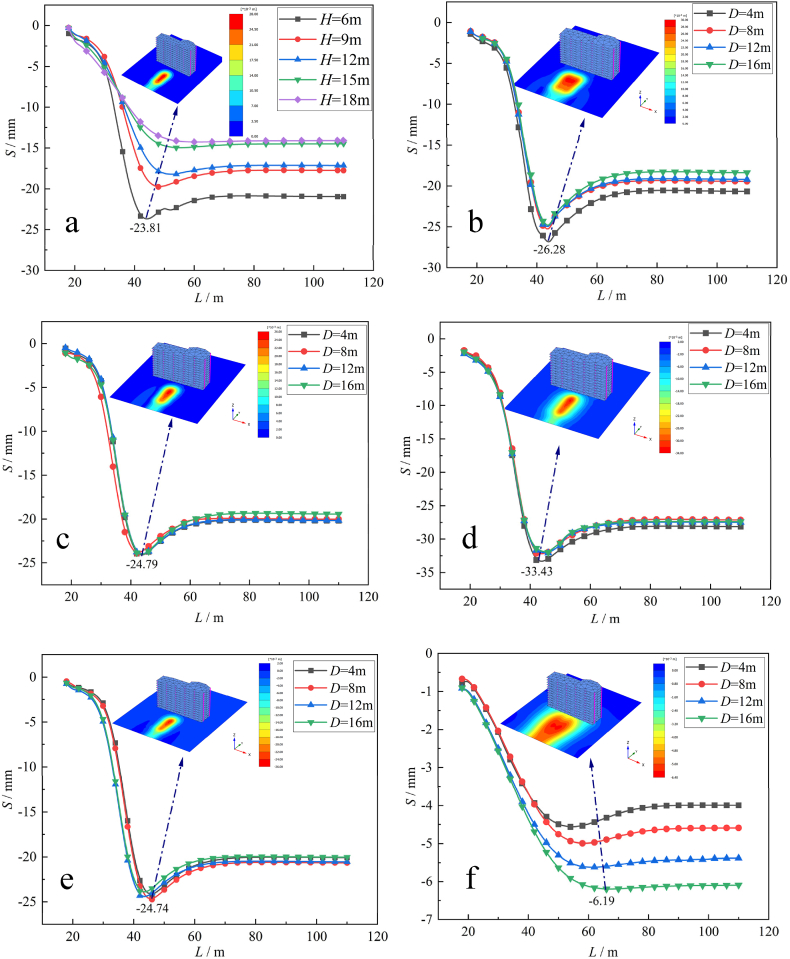
The L and *S* curves at different *H* under each working condition: (a) Single-line tunnel; (b) Left and right at the same time; (c) Left and right at the same time; (d) Up and down at the same time; (e) First down then up; (f) First up then down.

The simulation of double-track tunneling includes a vertical double tunnel and a horizontal double tunnel. Fig. 4 shows the relationship curve between the surface settlement *S* and shield tunneling distance *L* of the single-line tunnel and double-line tunnel under different *H*.

From Fig. 4(b), it can be seen that the maximum value of S under the simultaneous construction of two tunnels occurs when *D* = 4 m, which is −26.28 mm, and S decreases with increasing *D*. The settlement trough is generated by the superposition of two tunnels, and the influence range of surface settlement is larger than that of the single-line tunnel excavation in Fig. 4(a).

From Fig. 4(c), it can be seen that there is little difference in the working conditions *S* of the four different intervals in the construction of the double-line tunnel first left and then right, and the maximum surface settlement is −24.79 mm, which is slightly larger than that of the single-line tunnel driving.

From Fig. 4(d), it can be seen that when the two-line tunnel is constructed at the same time, the impact on the surface settlement is much greater than that caused by the single tunnel excavation, and the maximum value of *S* is −33.43 mm, which is 29.5 % higher than that of the single line tunnel excavation. The main reason for the *S* increase is that the soil loss on the tunnel axis caused by vertical double-line tunnel excavation is much greater than that of single-line tunnel excavation.

From Fig. 4(e) and (f), it can be seen that the maximum surface settlement of the double-track tunnel is −24.74 mm, and *h* has little influence on *S*. In the double-line tunnel excavation, S increases with increasing *h*, and the maximum surface settlement occurs when *h* = 8 m, which is −6.19 mm. Moreover, it can be clearly seen from the surface settlement cloud map that the surface settlement above the tunnel axis is smaller than that on both sides of the tunnel axis because the completion of the construction of the tunnel on the upper side will cause a “blocking effect” on the tunnel under construction. In other words, the soil displacement change caused by the excavation of the tunnel on the lower side will be blocked by the existing tunnel on the upper side so that the surface settlement will be reduced.

#### 3.1.2 Law of surface subsidence

**Fig. 5:**
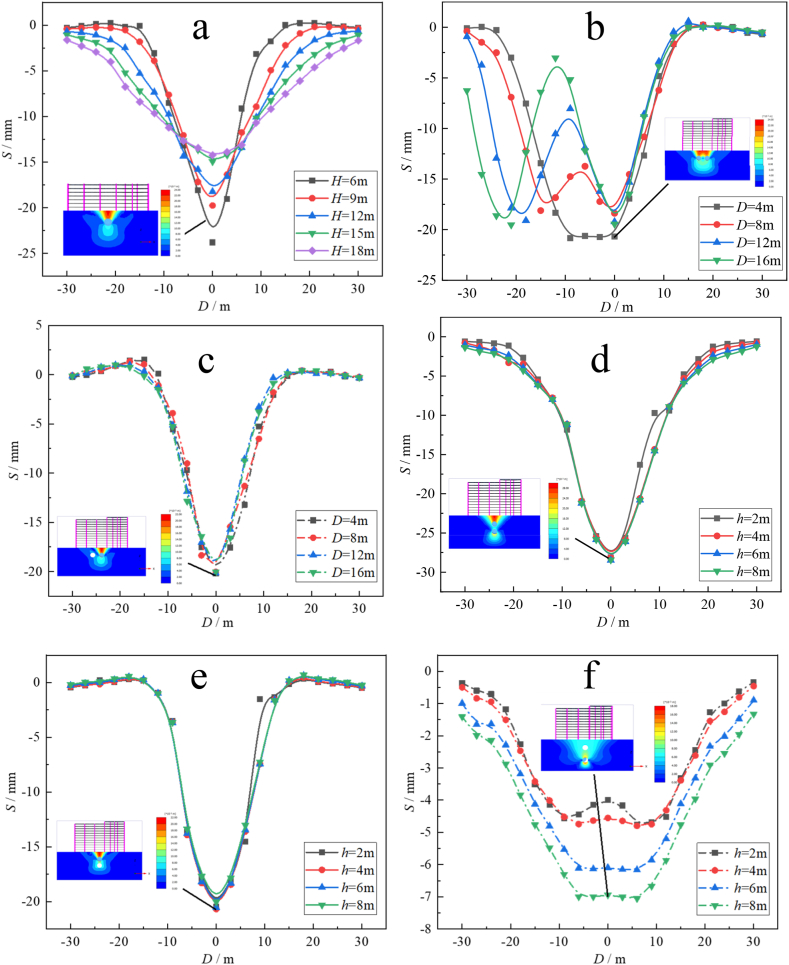
Settlement tank of the L = 35 m section under different working conditions: (a) Single-line tunnel; (b) Left and right at the same time; (c) First left then right; (d) Up and down at the same time; (e) First down then up; (f) First up then down.

Fig. 5 shows the settlement cloud of the horizontal section where monitoring point A is located. Compared with Fig. 5(a) shows that the settlement trough of the horizontal double-line tunnel in Fig. 5(b) presents a double-peak shape when it is constructed at the same time. As the tunnel spacing increases, the double-peak shape becomes more obvious, and the width of the settlement trough becomes larger.

From Fig. 5(c), it can be seen that when horizontal tunnels are constructed successively, the tunnel completed first will have a certain “blocking effect” on the postconstruction tunnel; that is, the influence of the tunnel constructed first on the side near the postconstruction tunnel is greater than that away from the side, the surface settlement on the left side of the settlement trough is slightly higher than that on the right side, and the surface settlement on the left side of the tunnel with the advance is less than the surface settlement on the right side of the existing tunnel without the advance.

Fig. 5(d), (e) and 5(f) show that the curve shape of the settling trough of the double-line tunnel under simultaneous construction up and down is similar to that of the single tunnel, but Smax is significantly larger than that of the single-line tunnel, and the width of the settling trough is slightly larger than that of the single-line tunnel under construction. The curve shape of the settlement trough of the double-track tunnel is also similar to that of the single-track tunnel. However, it can be seen from the displacement cloud map at the lower left corner of Fig. 5(e) that the soil disturbance on the side of the postconstruction tunnel facing the previous construction tunnel is significantly smaller than that on the other side. Therefore, the existence of the previous existing tunnel can affect the distribution of the disturbance area to some extent. From Fig. 5(f), it can be seen that for vertical double tunnels constructed in the sequence of first up and then down, due to the " blocking effect” of the upper tunnel, the maximum surface settlement is only −7.01 mm, which is obviously smaller than other working conditions. With the increase in the distance between two tunnels, S also increases. When *h* = 2 m, S on both sides of the tunnel axis is significantly larger than that directly above the axis.

As can be seen from Section 3.1, a two-lane tunnel requires more soil excavation during the boring process compared to a single-lane tunnel, resulting in greater ground loss and stress release, and thus greater surface settlement. Simultaneous excavation compared to sequential excavation will generate greater force on the ground surface, resulting in greater settlement, while sequential excavation can reduce the force on the ground surface because the tunnel excavated first can provide support for the tunnel excavated later, thus reducing the deformation of the ground surface, which is the same as expected.

The sequence of first up then down construction greatly reduced the surface settlement compared to the other construction sequences. However, in the study of tunnel spacing, the variation of horizontal spacing for sequentially excavated tunnels and vertical spacing for tunnels with up-and-down simultaneous and first-up-then-down construction sequences had insignificant effects in surface settlement. Moreover, in the tunnel with the construction sequence of first up then down, the surface settlement increases instead with the increase of spacing. This is not in accordance with the expectation, thus this section of the study is of exploratory and inspirational value.

### 3.2 Deformation law of buildings

#### 3.2.1 Building settlement

To study the influence of different spatial locations of the double-track tunnel on the settlement and deformation of the building, the influence of the double-track tunnel excavation on the settlement of the center point (monitoring point B) of the first floor of the building under different working conditions is plotted, as shown in Table 4.

**Table 3:** Grouting pressure value.

Tunnel depth *H*(m)	Minimum grouting pressure *P*_ma_(kN/m^2^)	Maximum grouting pressure *P*_up_(kN/m^2^)	Value of grouting pressure *P*(kN/m^2^)
6	71.43	200.18	120
9	110.39	292.57	160
12	149.36	384.96	200
15	188.32	477.35	250
18	227.29	569.75	300

**Table 4:** Settlement of monitoring point B during double tunnel construction under different working conditions.

Vertical spacing *h*	Up and down at the same time	First up then down	First down then up	Horizontal spacing *D*	Left and right at the same time	First left then right	Buried Depth *H*	single-line tunnel
2 m	−17.034	−5.6	−10.317	4 m	−16.009	−10.599	6 m	−10.812
4 m	−16.447	−5.365	−10.488	8 m	−13.666	−10.231	9 m	−11.215
6 m	−16.135	−5.300	−9.382	12 m	−12.489	−11.499	12 m	−11.734
8 m	−16.049	−5.685	−9.906	16 m	−11.806	−11.425		

Note: The settlement unit of the above monitoring points is mm.

By comparing the settlement table of monitoring point B during the construction of the two-line tunnel with that of the monitoring point at a 6 m buried depth of the single-line tunnel, it can be concluded that:

(1) The maximum settlement of the monitoring point of the building foundation under the construction condition of the vertical two-line tunnel is greater than that of the single-line tunnel construction scheme, the maximum settlement is at *h* = 2 m, and the settlement of the monitoring point decreases with increasing spacing. Due to the “blocking effect”, the settlement of monitoring point B is much smaller than that of the single-line tunnel. The influence of tunnel construction on building foundations is slightly less than that of single-line tunnel construction.
(2) The maximum settlement of the monitoring point of the building foundation is −16.009 mm when the distance *D* = 4 m is under the construction of the horizontal double-track tunnel, which is larger than the disturbance range caused by the construction of the single-track tunnel, resulting in the overall settlement of the building increasing by 26.7 %. The maximum settlement of monitoring point B caused by the first left and then right tunnel construction is −11.499 mm, which is similar to the maximum settlement value of the single line tunnel construction. However, the settlement of monitoring point B combined with the different spacings *D* is slightly smaller than that of the single line tunnel construction in general because the influence range of the postconstruction tunnel can be restricted after the previous tunnel construction is completed. Thus, the settlement of the building on the first side of the tunnel is reduced.

#### 3.2.2 Building inclination

**Fig. 6:**
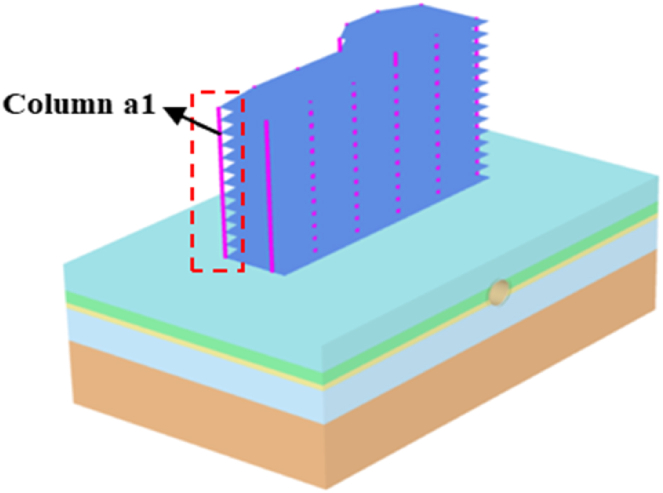
Diagram of the corner column of the building.

To explore the change law of the horizontal inclination value of the building (the ratio of the horizontal displacement difference generated by the top point and the bottom point of a certain position of the building to the height of the building) caused by the construction of the double-line tunnel under different working conditions, the maximum horizontal inclination ratio of the side corner column a1 of the building is taken as the monitoring object, as shown in Fig. 6. The results are shown in Table 5 and compared with the maximum horizontal inclination ratio of a single-line tunnel.

**Table 5:** Double tunnel a1 column horizontal tilt maximum ratio.

working condition (Vertical tunnel)	*h* = 2 m	*h* = 4 m	*h* = 6 m	*h* = 8 m
Up and down at the same time	0.024 %	0.025 %	0.031 %	0.031 %
first down then up	0.017 %	0.017 %	0.018 %	0.017 %
first up then down	0.011 %	0.01 %	0.009 %	0.009 %
working condition (Horizontal tunnel)	*D* = 4 m	*D* = 8 m	*D* = 12 m	*D* = 16 m
First left then right	0.019 %	0.016 %	0.020 %	0.017 %
Left and right at the same time	0.041 %	0.058 %	0.062 %	0.066 %

As seen from Table 5, the maximum inclination value of column a1 of the double tunnel constructed at the same time is 0.031 %, which is significantly greater than that of the single-line tunnel *H* = 6 m (0.02 %), and the inclination value increases with the increase in the vertical distance of the tunnel. In the case of the existing tunnel on the lower side, the inclination value of column a1 of the building is less than that in the case of the single-line tunnel, but the tunnel spacing has little influence on the inclination value. Under the condition of the existing tunnel on the upper side, the maximum tilt ratio of column a1 is significantly smaller than that of the single-line tunnel. In this case, due to the “blocking effect”, the influence of the postconstruction tunnel on the building on the lower side is less, and the minimum tilt value is only 0.009 %, which is 55 % lower than that of the single tunnel.

In the condition of a horizontal double tunnel, the inclined result of column a1 caused by the first left and then right double tunnels is similar to that of a single tunnel, approximately 0.02 %. However, the settlement of column a1 is significantly greater than that of single-line tunnel construction under the condition of left and right simultaneous double tunnels. The greater *D is*, the greater the inclination, but the increasing trend tends to be gentle, and the maximum inclination is 0.066 % when *D* = 16 m.

### 3.3 Stress variation law

To explore the law of the influence of the double-line tunnel underpass on the internal forces of the overlying building, Table 6, Table 7 list the maximum axial force, shear force and bending moment of the plate and column structure of the building during the construction of the horizontal double-line tunnel under different working conditions.

**Table 6:** Internal forces of the building plate structure under different working conditions of the horizontal double tunnel.

	Horizontal spacing	Axial force(kN)	Shear force(kN)	Bending moment (kN·m)
First left then right	*D* = 4 m	1320.2	867.2	395.9
	*D* = 8 m	1270.6	942.6	368.9
	*D* = 12 m	1316.7	946.5	371.8
	*D* = 16 m	1272.1	924.5	369.7
Left and right at the same time	*D* = 4 m	1364.6	932.2	417.5
	*D* = 8 m	1278.3	1050.7	369.1
	*D* = 12 m	1214.2	997.4	392.3
	*D* = 16 m	1194.2	916.3	409.5
	No Tunnel	880.7	786.3	367.6

**Table 7:** Internal forces of the building column structure under different working conditions of the horizontal double tunnel.

	Horizontal spacing	Axial force(kN)	Shear force(kN)	Bending moment (kN·m)
First left then right	*D* = 4 m	5008.6	363.3	1008.5
	*D* = 8 m	5007.6	437.5	971.2
	*D* = 12 m	5007.6	445.1	1013.1
	*D* = 16 m	5007.4	468.6	1011.4
Left and right at the same time	*D* = 4 m	5008.6	442	1087.9
	*D* = 8 m	5006.6	484.9	1021.7
	*D* = 12 m	5007.3	477.5	1064.5
	*D* = 16 m	5008.4	486.6	1009.8
	No Tunnel	4991	315.9	824.7

It can be seen from Table 6 that during the construction of double tunnels, the influence of simultaneous construction on the internal forces of the plate structure of the building is as follows: axial force > shear force > bending moment, in which the maximum value of axial force is approximately 4 m distance between the two tunnels simultaneously constructed, which is 1366.6 kN; the maximum value of shear force is approximately 8 m distance between the two tunnels simultaneously constructed, which is 1050.7 kN. The maximum bending moment is approximately 4 m between the two tunnels at the same time, so it can be concluded that the influence of a small distance between the two tunnels on the internal forces of the plate structure is slightly greater than that of a large distance. Table 7 The internal forces of the column structure of the building without a tunnel changes little compared with other conditions, and the bending moment and shear change are more obvious.

**Fig. 7:**
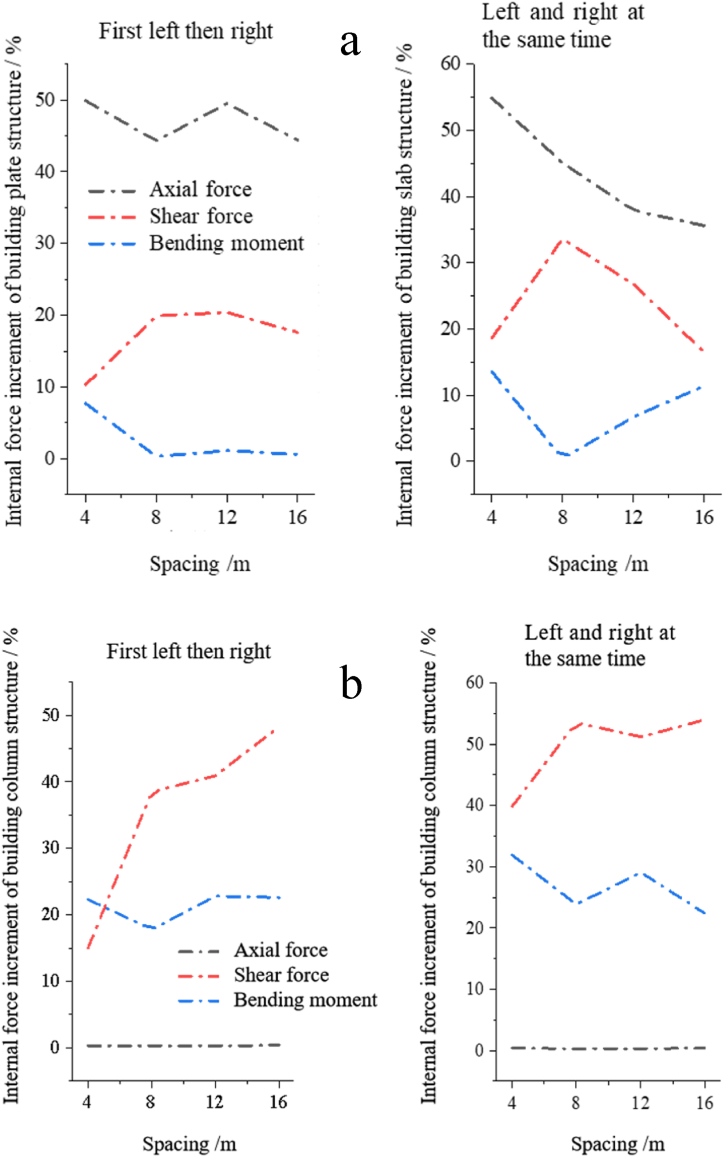
Under the change curve of the internal forces increment in the building plate and column structure of the horizontal double tunnel: (a) Internal forces of plate structure; (b) Internal forces of the column structure.

To further explore the relationship between the internal forces of the building and the tunnel spacing, the maximum axial force, shear force and bending moment increment of the plate and column structure of the building with *H* are plotted, as shown in Fig. 7.

From Fig. 7(a) and (b), it can be seen that the internal forces of buildings under different spacings are similar to those under left-to-right construction and left-to-right construction at the same time. In the plate structure, the axial force increment > shear force increment > bending moment increment; in the column structure, the shear force increment > bending moment increment > axial force increment. The change in axial force mainly occurs in the plate structure, while the change in shear force and bending moment occurs in the column structure. In left-right simultaneous construction, with the increase of tunnel horizontal spacing, the moment increment and shear force increment of the plate and column are negatively correlated, the axial force increment of the plate decreases gradually and the shear force increment of the column increases gradually; In the first left then right construction, with the increase of the horizontal spacing of the tunnel, the bending moment increment and shear force increment of the plate were negatively correlated, the axial force increment of the plate had no significant deformation trend, and the shear force increment of the column gradually increased. Therefore, it is necessary to increase the supporting and fixing devices to reduce the shear force increment of the column structure and the axial force increment of the plate structure.

As seen from Table 8, Table 9, the internal forces of the vertical double-line tunnel plate structure are as follows: double tunnel at the same time up and down > double tunnel at the same time up and down > double tunnel at the same time up and down > no tunnel. With increasing spacing, the axial force, shear force and bending moment of the plate and column structure of the building show a decreasing trend, among which the maximum axial force of the plate structure is 1365.7 kN, the maximum shear force is 1031.6 kN, the maximum bending moment is 400.6 kN m, the maximum axial force of the column structure is 5012.6 kN, and the maximum shear force is 474.5 kN. The maximum bending moment is 977.2 kN, and the above maximum internal force occurs in the condition of simultaneous construction on both sides of the double tunnel with a distance of 2 m.

**Table 8:** Internal forces of the building plate structure of the vertical double tunnel under different working conditions.

	Vertical spacing	Axial force(kN)	Shear force(kN)	Bending moment (kN·m)
Up and down at the same time	*h* = 2 m	1365.7	1031.6	400.6
	*h* = 4 m	1340.3	1019.2	397.3
	*h* = 6 m	1316.3	1001.3	396.6
	*h* = 8 m	1298.4	974.5	394.9
First up then down	*h* = 2 m	932.6	827.3	373.5
	*h* = 4 m	942.9	816.9	372.6
	*h* = 6 m	926.7	801.3	371.6
	*h* = 8 m	927.3	799.7	371.4
First down then up	*h* = 2 m	1281.9	970.2	390.8
	*h* = 4 m	1283.8	976.4	390.9
	*h* = 6 m	1264.2	956.1	391.9
	*h* = 8 m	1261.3	947.8	390.8
	No Tunnel	880.7	786.3	367.6

**Table 9:** Internal forces of the building column structure of the vertical double tunnel under different working conditions.

	Vertical spacing	Axial force(kN)	Shear force(kN)	Bending moment (kN·m)
Up and down at the same time	*h* = 2 m	5012.6	474.5	977.2
	*h* = 4 m	5012.6	457.8	963.8
	*h* = 6 m	5009.6	453	944.9
	*h* = 8 m	5010.4	447.1	924.1
First up then down	*h* = 2 m	4994.9	343.9	824.7
	*h* = 4 m	4993.3	339.4	832.7
	*h* = 6 m	4992	340	825.1
	*h* = 8 m	4994.9	342	824.4
First down then up	*h* = 2 m	5007.7	404	970.5
	*h* = 4 m	5008.5	407.8	973.5
	*h* = 6 m	5007.7	415.6	963.2
	*h* = 8 m	5007.7	417.8	963.6
	No Tunnel	4991	315.9	824.7

**Fig. 8:**
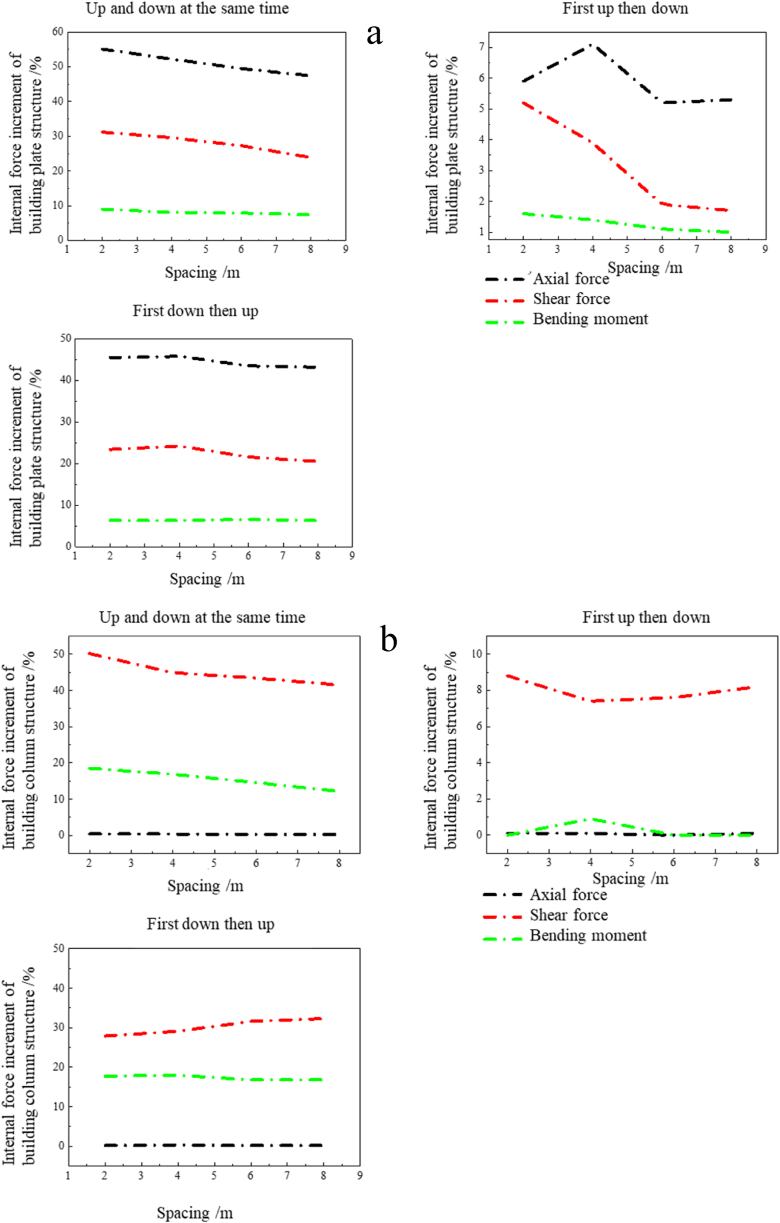
Curve of the internal forces increment of the building plate and column structure under a vertical double-line tunnel: (a) Internal forces of plate structure; (b) Internal forces of the column structure.

Fig. 8 shows the variation curve of the maximum axial force, shear force and bending moment increment of the plate and column structure of the vertical double-line tunnel under the tunnel with *h*.

From Fig. 8(a) and (b), it can be seen that in the increment of the internal forces of the plate structure of the building, axial force > shear force > bending moment. In the internal forces increment of the building column structure, shear force > bending moment > axial force. Regardless of the column structure or plate structure of the building, the internal forces increment is the largest under the construction condition at the same time, followed by the internal forces increment under the construction condition first and then, and the internal forces increment is the smallest when the construction sequence is first and then. The change in the internal forces of the overlying building in the underpass tunnel also conforms to the influence law of the “disturbance effect” and “blocking effect” on the surface settlement in the aforementioned double-line tunnel construction.

In the up-and-down simultaneous construction and the down-first-back-up construction, there is no significant variable trend of internal forces with the increase of vertical spacing; however, in the up-first-back-down construction sequence, the increment of internal forces decreases dramatically compared to the other two, and the axial force and shear force of the plate decreases with the increase of vertical spacing. Therefore, the construction sequence of up first then down is prioritized to be used in vertical two-lane tunnel construction with increasing spacing appropriately to minimize impacts on the building.

In summary, it can be seen from the simulation results of each scheme that the spatial effect of the two-lane tunnel construction significantly affects the surface settlement and the deformation of the buildings and structural internal forces. The “blocking effect” or “disturbance effect” generated by the prior constructed tunnels on the later constructed tunnels can affect the distribution of the disturbed area to a certain extent. When the “blocking effect” is generated, the surface settlement, building deformation and internal forces increment are significantly reduced, which has obvious deformation control advantages. The law of change of internal forces in the building during the construction of a two-lane tunnel is similar to that of single-lane tunnel, with changes in axial forces occurring mainly in the slab structure and changes in shear forces and bending moments occurring in the column structure of the building.

In addition to minimizing the disturbance to the overlying soil and buildings, shield tunnel construction should also meet the safety requirements of the tunnel structure itself. Therefore, the next step of the study will be to carry out the research on the engineering influence law from the safety of the tunnel structure.

## 4 Engineering influence law

### 4.1 Engineering influence zoning

#### 4.1.1 Engineering impact zoning theory

Disturbance impact zoning of shield construction is a spatial zoning of the extent of construction impacts on neighboring structures according to specific criteria [38]. In this paper, the impact level is determined by the ratio of the safety factor with and without building loads, the ratio of safety factors ξ = *β*_*1*_/*β*_*0*_. The 7, 10 and 13-story buildings were simulated using loads of 0.27 MPa, 0.38 MPa and 0.5 MPa, respectively. The formula for the factor of safety based on the strength discount method is as Eq. (9):

(9) $$F_{s}=S_{\max}/S_{balance}$$

Where *F*_s_ is the safety factor; *S*_max_ ann *S*_balance_ are the maximum shear strength and the shear stress in equilibrium, respectively.

By importing the Mohr-Coulomb criterion, the safety factor can also be expressed as Eq. (10):

(10) $$F=\left(c-\sigma_{n}\;\tan\;\varphi \right)/\left(\sigma_{n}\;\tan\;\varphi_{r}\right)$$

### 4.2 Safety influence law of double-track tunnels

#### 4.2.1 Construction of a two-lane tunnel at the same time

Table 10 shows the change in the safety factor and its ratio ξ of double-line tunnels under simultaneous construction. It can be seen from the table that when there is no building load, the safety factor values of the double-line tunnel simultaneously constructed at *D* = 12 m and 16 m are basically the same. The smaller the two-track tunnel spacing is, the smaller the safety factor. When *D* = 4 m, the safety factor is 4.785. When there is a building load, the safety factor gradually decreases with increasing load, and the safety factor is 2.623 at 0.27 MPa and 1.964 at 0.5 MPa. At the same time, when *D* = 4 m, the safety factor ratio *ξ* is significantly smaller than other spacings, and when *D* ≥ 8 m, *ξ* increases slowly with increasing spacing until it becomes stable. Therefore, it can be considered that the ratio of the safety factor changes obviously when there is no building in the range of *D* ≤ 8 m. At this time, as the distance between the two tunnels approaches, the range of the plastic zone of the surrounding rock increases, and the probability of tunnel instability risk increases.

**Table 10:** Safety factor and ratio ξ of the two-lane tunnel under the left and right sides at the same time.

Horizontal spacing (*D*)	4 m		8 m		12 m		16 m	
No building load	4.785		4.889		5.019		5.018	
0.27 MPa	2.623	54.8 %	2.968	60.7 %	2.973	59.2 %	2.996	59.7 %
0.38 MPa	2.229	46.6 %	2.529	51.7 %	2.530	50.4 %	2.539	50.6 %
0.5 MPa	1.964	41.0 %	2.190	44.8 %	2.206	43.9 %	2.249	44.8 %

#### 4.2.2 Existing tunnel on the left side two-lane tunnel

Table 11 shows the safety factor and its ratio ξ change of the existing tunnel on the left. It can be seen from the table that the safety factor under this working condition does not change significantly with increasing horizontal distance *D*, and the safety factor basically tends to a fixed value within the simulation range of 4 m ≤ *D* ≤ 16 m. This is because the left tunnel has been completed, the stress state of the existing tunnel has been stabilized, the left tunnel safety reserve is sufficient, and the change in horizontal distance *D* has little influence on the safety factor of the existing tunnel. With the increase in the building load, the safety factor and its ratio gradually decrease. In the case of the same burial depth, the ratio *ξ* has no obvious correlation with the horizontal distance *D*. In general, the safety factor and its ratio of the existing tunnel on the left side are higher than that of the two-lane tunnel constructed at the same time.

**Table 11:** Safety factor and its ratio ξ of two-lane tunnel under first left then right.

Horizontal spacing (*D*)	4 m		8 m		12 m		16 m	
No building load	5.036		5.010		5.091		4.969	
0.27 MPa	2.967	58.9 %	3.053	60.9 %	3.010	59.1 %	3.024	60.9 %
0.38 MPa	2.517	49.9 %	2.576	51.4 %	2.559	50.3 %	2.568	51.7 %
0.5 MPa	2.204	43.8 %	2.233	44.6 %	2.253	44.3 %	2.285	45.9 %

#### 4.2.3 Construction of a two-line tunnel simultaneously

Table 12 shows the safety factor and its ratio ξ change of the double-line tunnel under construction at the same time. As seen from the table, with the increase in vertical spacing, the safety factor decreases. In the case of no building load, the safety factor is 3.078 when *H* = 2 m and 2.484 when *H* = 8 m. The safety factor of the double-track tunnel constructed at the same time is lower than that of the double-track tunnel constructed at the same time. With the increase in the overlying building load, the safety factor and its ratio gradually decrease. The overlying load is the main factor affecting tunnel construction safety. At the same time, as the distance *H* between the upper and lower tunnels increases, the ratio of the safety factor with or without buildings becomes increasingly larger, and the influence of the load on tunnel stability decreases, indicating that the tunnel distance *H* affects tunnel safety to a certain extent. The main reason for tunnel instability is that the tunnel itself is buried too deep.

**Table 12:** Safety factor and its ratio ξ of a two-lane tunnel under up and down at the same time.

Vertical spacing (*H*)	2 m		4 m		6 m		8 m	
No building load	3.078		2.876		2.611		2.484	
0.27 MPa	2.718	88.3 %	2.681	93.2 %	2.469	94.6 %	2.380	95.8 %
0.38 MPa	2.581	83.8 %	2.519	87.6 %	2.420	92.7 %	2.347	94.5 %
0.5 MPa	2.221	72.2 %	2.249	78.2 %	2.258	86.5 %	2.277	91.7 %

#### 4.2.4 Existing tunnel on the lower side of the two-lane tunnel

Table 13 shows the safety factor and its ratio ξ change of the existing tunnel on the lower side. As seen from the table, as the overlying load increases from 0.27 MPa to 0.5 MPa, the safety factor decreases. When the load is 0.27 MPa, the safety factor is approximately 3; when the load is 0.38 MPa, the safety factor is between 2.5 and 2.6; when the load is 0.5 MPa, the safety factor decreases between 2.2 and 2.3. With the increase in the distance *H* between the two tunnels, the safety factor basically does not change, so it can be seen that the value of the safety factor in this case is only related to the location of the tunnel on the upper side and the load of the building. Meanwhile, the *ξ* value of the same building load is almost the same, but they are all smaller than the simultaneous construction of the double-line tunnel under the same working conditions, indicating that the influence of the building load on the construction of the double-line tunnel under the existing tunnel is higher than that of the simultaneous construction of the double-line tunnel.

**Table 13:** Safety factor and its ratio *ξ* of a two-lane tunnel under first down and then up.

Vertical spacing (*H*)	2 m		4 m		6 m		8 m	
No building load	4.995		5.059		4.944		4.953	
0.27 MPa	3.032	60.7 %	3.029	59.9 %	3.025	61.2 %	3.004	60.7 %
0.38 MPa	2.571	51.5 %	2.551	50.4 %	2.589	52.4 %	2.583	52.2 %
0.5 MPa	2.232	44.7 %	2.231	44.1 %	2.257	45.6 %	2.242	45.3 %

#### 4.2.5 Existing tunnel on the upper side two-lane tunnel

Table 14 shows the safety factor and its ratio *ξ* change of the existing tunnel on the upper side. As seen from the table, when there is no building load, the safety coefficient of the existing double-track tunnel on the upper side is almost the same as that of the double-track tunnel under simultaneous construction on the upper and lower sides. When *H* = 2 m, the safety coefficient is 3.053, and when *H* = 8 m, the safety coefficient is 2.439. With the increase in building load, the safety factor gradually decreases, but the reduction amplitude is obviously smaller than that of the double-line tunnel under construction at the same time because the completed tunnel on the upper side has a blocking effect on the tunnel under construction on the lower side. To a certain extent, the upper tunnel blocks the influence of the surface building load on the lower tunnel. As the distance between the two tunnels increases from 2 m to 8 m, the safety factor decreases with increasing burial depth. When the overlying building load is 0.5 MPa and the distance between the two tunnels is 8 m, the overall safety factor of the model is 2.419, which is slightly higher than the other two double-tunnel conditions.

**Table 14:** Safety factor and its ratio ξ of a two-lane tunnel under first up then down.

Vertical spacing (*H*)	2 m		4 m		6 m		8 m	
No building load	3.053		2.901		2.641		2.439	
0.27 MPa	2.803	91.8 %	2.740	94.4 %	2.638	99.9 %	2.433	99.8 %
0.38 MPa	2.653	86.9 %	2.611	90.0 %	2.524	95.6 %	2.429	99.6 %
0.5 MPa	2.571	84.2 %	2.548	87.8 %	2.520	95.4 %	2.419	99.2 %

At the same time, the *ξ* value of the existing double-track tunnel construction on the upper side is higher than that of the other two double-track tunnels. When the distance between the two tunnels is 2 m and the load is 0.5 MPa, the safety factor ratio is the smallest, which is 84.2 %. When the distance between two tunnels exceeds 6 m, the safety factor ratio ξ approaches 1, and the influence of the building load on the tunnel under construction is almost the same.

In summary, with the increase of construction load, the safety factor and the ratio gradually decrease, but the magnitude of the decrease gradually decreases, and the safety factor are kept above 1.9, all have a certain safety reserve. However, as can be seen from Table 10, Table 11, Table 12, Table 13, Table 14, the main factors in the engineering influence zoning are the spatial location of the tunnels and the construction sequence. The ratio of the safety factor for horizontal double tunnels is smaller compared to that for vertical double tunnels. In vertical double tunnels, the ratio of the safety factor for tunnels constructed first down and then up is significantly smaller than that for tunnels constructed both up and down simultaneously and first up and then down, and the ratio increases with the increase of the vertical spacing, reaching or approaching the no-impact zone. For the above study, the following practical guidelines are proposed:

(1) When planning tunnel paths, important facilities, such as other underground utilities, dense traffic areas above ground, and buildings, should be avoided as much as possible.
(2) In the spatial layout, if the tunnel needs to pass underneath a building, geological investigation should be carried out in detail, the type of building piles should be understood, priority should be given to the vertical construction sequence of first up then down, and appropriate protection measures should be taken, including setting up supports for the building underneath, reinforcing the foundation of the building, and controlling the construction loads.
(3) When planning multiple shield tunnels, tunnel spacing should be maintained appropriately to prevent mutual interference. At the same time, the impact of tunnel construction on the surrounding soil layer and the interaction between tunnels should be considered.

## 5 Discussion

For the safety hazard of shield tunnels passing through buildings, we have carried out a lot of work, including refined 3D numerical simulations based on the site conditions, analyzing the surface deformation, building deformation, and internal forces changes, and identifying the causes based on the results, and proposing the corresponding construction suggestions. Compared with other research results, we focus on the spatial effects of the two-lane tunnels and the internal forces in the load-bearing structures of the buildings. This study reveals the effects of different construction sequences, tunnel spacing and other factors on surface settlement and internal forces changes of buildings, especially the application of the blocking effect. Engineers can choose more rational construction sequences and tunnel layouts based on these findings to minimize the adverse effects on the ground surface and buildings. The application of safety coefficient and ratio variation in this study can help designers to consider various spatial effects at the planning stage and propose targeted protection measures to avoid potential engineering risks. Overall, this study has certain reference value for the prevention of ground subsidence, building tilting and cracking. Since subways are constructed in large numbers worldwide and traverse urban areas, this research can be studied in greater depth in the future in a number of aspects, such as traversing geologically complex water-rich rounded gravel pebble layers, integration construction of shield tunnels, special shaped tunnels, and traversing dense building clusters, among others.

## 6 Conclusion

In this paper, two-line shield tunnel simulations were carried out under different vertical spacings *D*, horizontal spacings *H*, construction sequences and building floors. This paper analyses the influence of the space effect of double-line tunnel shield tunneling on the surface and buildings from four aspects: surface settlement, building settlement deformation, building stress change and influence law, and draws the following conclusions:

(1) Two-lane shield tunnels with different tunnel spacing and construction sequences have significant deformation effects on the ground surface compared to single-lane tunnels. Smaller tunnel spacing leads to greater surface settlement. The maximum surface settlement occurs in the up and down at the same time construction of the two-lane tunnel, which is −33.43 mm, and the minimum surface settlement occurs in the case of the first up then down construction, which is −6.19 mm, and the horizontal construction tunnels do not have a significant difference in the effect of the surface settlements. In the process of two tunnel construction, the “blocking effect” produced by the previous construction tunnel on the later construction tunnel can affect the distribution of the disturbed area to a certain extent, and reduce the deformation of the ground surface settlement.
(2) For building settlement, simultaneous construction > single tunnel > sequential construction, and the deformation of the building monitoring points decreases as the spacing increases. The maximum value of the monitoring point is 17.034 mm in the up and down at the same time, and 16.009 mm in the left and right at the same time, both larger than the single tunnel construction. The first construction tunnel has a “blocking effect” on the later construction tunnel, so the sequential construction tunnel has less impact on the building. Among them, the settlement of the tunnel construction in the order of first up and then down is much smaller than that of the single-lane tunnel, which is 5.30 mm.
(3) In the inclined analysis of the vertical double-line tunnel to the building, the maximum inclined value of column a1 is 0.031 % for the simultaneous construction of thetwo-line tunnel. In the case of the existing tunnel on the lower side, the inclination value of column a1 of the building is slightly smaller than that of the single-line tunnel. The maximum tilt ratio of column a1 in the existing tunnel on the upper side is the smallestl, and the minimum tilt value is only 0.009 %. In a horizontal two-lane tunnel, the inclined result of column a1 of the building caused by the construction of the first left and then right is similar to that of the single tunnel, but the settlement of column a1 is significantly larger than that of the single tunnel under the construction of the left and right at the same time, and the greater *D is*, the greater the inclination.
(4) During the construction of the double-line tunnel, the internal forces of the building changes as follows: axial force increment > shear force increment > bending moment increment in plate structure, shear force increment > bending moment increment > axial force increment in column structure. The change in axial force mainly occurs in the plate structure, while the change in shear force and bending moment occurs in the column structure. The construction sequence of first up then down has the least effect on the incremental internal forces in the structure.
(5) The changes in the safety factor and safety factor ratio *ξ* in the five different working conditions of the double-line shield tunnel are also different. The safety factor value of the upper and lower double-track tunnels is generally greater than that of the left and right double-track tunnels. The construction safety factor of the double-track tunnel under the existing tunnel is greater than that of the double-track tunnel under simultaneous construction. Under normal circumstances, the smaller the distance between the two tunnels is, the lower the safety factor, and with the increase in the overlying building load, the safety factor will also decrease. Among them, the factor of safety and its ratio for the two-lane tunnel with the first up then down construction sequence are better than the other conditions, and the ratio is more than 99 % for the spacing h = 8m, which is almost indistinguishable from the factor of safety under no construction loads.
(6) In the actual project, the numerical simulation also needs to consider the influence of different locations of multiple buildings, vehicle loads, underground seepage and other complex environments. These are important factors affecting shield tunneling and the future application of rectangular shields in subways, for which further research can be carried out in the future.

## Ethics statement

Review and/or approval by an ethics committee was not needed for this study because this is an article about engineering risk assessment and analysis, there are no ethical issues involved. For the same reason, informed consent was not required.

## Funding and acknowledgments

This work is supported by the 10.13039/501100004607Guangxi Natural Science Foundation (2022GXNSFBA035580).

## Data availability statement

Data will be made available on request.

## Additional information

No additional information is available for this paper.

## CRediT authorship contribution statement

**Zhiqiang Chen:** Resources, Project administration, Investigation. **Wenlong Xiang:** Supervision, Resources, Investigation. **Zhaojian Hu:** Writing – original draft, Visualization, Validation, Data curation. **Mingjin Li:** Supervision, Methodology. **Jintao Wang:** Resources, Methodology, Investigation. **Dongxiang Hou:** Writing – original draft, Software, Formal analysis, Conceptualization. **Zhen Huang:** Writing – review & editing, Supervision, Funding acquisition, Conceptualization.

## Declaration of competing interest

The authors declare that they have no known competing financial interests or personal relationships that could have appeared to influence the work reported in this paper.
